# On the need for monitor unit calculations as part of a beam commissioning methodology for a radiation treatment planning system

**DOI:** 10.1120/jacmp.v1i3.2640

**Published:** 2000-06-01

**Authors:** George Starkschall, Roy E. Steadham, Nathan H. Wells, Laura O'Neill, Linda A. Miller, Isaac I. Rosen

**Affiliations:** ^1^ Department of Radiation Physics The University of Texas M. D. Anderson Cancer Center Houston Texas 77030‐4095

**Keywords:** monitor unit calculations, photons, treatment planning, beam commissioning

## Abstract

This paper illustrates the need for validating the calculation of monitor units as part of the process of commissioning a photon beam model in a radiation treatment planning system. Examples are provided in which this validation identified subtle errors, either in the dose model or in the implementation of the dose algorithm. These errors would not have been detected if the commissioning process only compared relative dose distributions. A set of beam configurations, with varying field sizes, source‐to‐skin distances, wedges, and blocking, were established to validate monitor unit calculations for two different beam models in two different radiation treatment planning systems. Monitor units calculated using the treatment planning systems were compared with monitor units calculated from point dose calculations from tissue‐maximum ratio (TMR) tables. When discrepancies occurred, the dose models and the code were analyzed to identify the causes of the discrepancies. Discrepancies in monitor unit calculations were both significant (up to 5%) and systematic. Analysis of the dose computation software found: (1) a coordinate system transformation error, (2) mishandling of dose‐spread arrays, (3) differences between dose calculations in the commissioning software and the planning software, and (4) shortcomings in modeling of head scatter. Corrections were made in the beam calculation software or in the data sets to overcome these discrepancies. Consequently, we recommend incorporating validation of monitor unit calculations as part of a photon beam commissioning process.

PACS number(s): 87.53.–j, 87.66.–a

## INTRODUCTION

Beam commissioning is the process by which parameters are determined for a beam model in a radiation treatment planning system and validated to assess how well the model and parameters reproduce measured dose distributions. Depending on the complexity of the beam model, the commissioning process may be simply a verification that the data has been input correctly, or it may be a complicated procedure of parameter fitting. Regardless of the complexity of the process, the major component of beam commissioning compares dose distributions calculated via the treatment planning system with measured dose distributions.

A component of the beam commissioning process that is often overlooked is the assessment of how well the treatment planning system calculates monitor units for beam delivery. This is not unusual, because monitor units are often determined outside the treatment planning system and based on the geometric set up of the patient as well as the dose prescription. As long as the treatment planning system accurately calculates relative dose distributions, one typically can obtain an absolute dose from an independent monitor unit calculation.

On the other hand, the ability to calculate monitor units accurately should be an essential component of the commissioning of a beam model in a treatment planning system. Good clinical practice requires that monitor unit calculations be checked independently, and the calculation of monitor units by the treatment planning system can provide this independent check. For dynamic therapies such as dynamic multileaf collimation, the calculation by the treatment planning system may be the only source of monitor units. Finally, the calculation of monitor units in a treatment planning system can be a sensitive indicator of errors in the treatment planning system, either in the dose calculation algorithm or in the specific manner in which the algorithm is implemented.

This paper addresses the last of these three arguments for validation of monitor unit calculations. In this paper, we illustrate four examples of situations in which discrepancies in monitor unit calculations, determined during the beam commissioning process, led to identification of errors in the computation of dose in the treatment planning system. In one example, using an in‐house treatment planning system, we found that monitor units, correctly computed at a 100‐cm source‐to‐skin distance (SSD), were systematically in error at other SSD values. This discovery led us to identify a problem in the coordinate system in which beam doses were calculated. In a second case, we found that monitor units in a commercial treatment planning system were calculated differently depending on the order in which beams were computed. After the manufacturer was notified of this problem, they identified an error in the manner in which dose‐spread arrays were stored for photon convolution calculations. In a third case, we found a small but systematic discrepancy in the monitor unit calculation. Analysis of the methods in which beams were calculated led us to conjecture that slightly different approximations were used in the calculations used for beam commissioning from approximations used in treatment planning. A small correction in the beam output corrected this discrepancy. A fourth case, examining monitor units for elongated fields, illustrated possible shortcomings in the way a beam model handled head scatter. Modifications were made in the parameters used to describe this beam component. These modifications compromised slightly on the fit of relative dose distributions but provided a much better monitor unit calculation.

It should be emphasized that none of the subsequent discussion is meant to reflect poorly on either the algorithms used in the dose modeling or on the treatment‐planning systems. Once the dose computation code had been modified or work arounds had been established, both treatment‐planning systems were used confidently in our clinic. The significant point is that a comparison of monitor unit calculations is a very sensitive tool for identifying errors either in the dose computation algorithm or the implementation of the algorithm. These errors, although small, are nonetheless errors and need to be identified and corrected and validation of monitor unit calculations should be considered an essential part of the commissioning process for a treatment planning system even if the monitor unit calculations are not used clinically.

## METHODS

### Relation of calculated values to true machine output

Dose calculation algorithms generally compute relative doses normalized to a reference value. If the dose calculation algorithm can relate the reference dose to a measured dose, it is capable of computing monitor units. This study examines two dose‐calculation algorithms. The first algorithm is the fast Fourier transform (FFT) convolution algorithm of Boyer *et al*.,^1^ ^,^ ^2^ as implemented in COPPERPlan, the in‐house radiation treatment planning system developed at The University of Texas M.D. Anderson Cancer Center.^3^ The second algorithm is the convolution/superposition algorithm of Mackie *et al*.^4^ ^,^ ^5^ as implemented in Pinnacle^3^ (ADAC Laboratories, Inc., Milpitas CA), a commercial radiation treatment planning system. Both algorithms are based on the model that the dose can be expressed as the convolution of incident photon fluence with a dose‐spread kernel as indicated in Eq. (1) as follows:

(1) $$D(r)=\int_{E}dE\int_{V}d^{3}r^{\prime }\frac{\mu }{\rho }(r^{\prime },E)\frac{d\Psi (r^{\prime },E)}{dE}A(r-r^{\prime },E),$$

where $\mu /\rho (r^{\prime },E)$ is the mass attenuation coefficient, $d\Psi (r^{\prime },E)/dE$ is the energy fluence spectrum at position $r^{\prime }$, and $A(r-r^{\prime },E)$ is the convolution kernel. Although both models begin with the same equation, the implementation of the two models is significantly different, as is the method by which the reference doses are related to measured quantities.

#### FFT convolution algorithm

A component of the process of commissioning the beam model for the COPPERPlan implementation of the FFT convolution algorithm is to scale the in‐air fluence by a normalization factor. Normalization factors are determined for a set of field sizes by matching calculated photon doses to measured doses at a specified reference depth (typically $d_{\max}$) and reference SSD (typically 100 cm) for a set of square fields defined by the collimators. For field sizes intermediate to those used in determining the normalization factors, the in‐air fluence is interpolated between the calculated values. In the presence of blocks, wedges, or other beam modifiers, the in‐air fluence is attenuated as the beam passes through the modifiers. Thus for a specified value of in‐air fluence, the absolute dose delivered to a calculation point will be reduced due to beam attenuation. The in‐air fluence is also modified due to beam divergence, accurately reflecting the distance of the calculation point from the source.

As a consequence of this modeling, photon dose distributions are represented in COPPERPlan relative to the dose delivered to the reference depth at the reference SSD for the unmodified photon field defined by the collimators of the treatment machine. We will refer to this field as the “reference field.” When the planner specifies a dose delivered by the beam to isocenter (“isocentric weight”), the treatment‐planning system returns a quantity termed the “beam weight.” This beam weight is the dose delivered to central‐axis $d_{\max}$ by the reference field for which the monitor units would deliver the same dose by the treatment field to isocenter. Thus in order to compute monitor units for a treatment field, the planner computes monitor units required to deliver the dose specified by the beam weight for the reference field.

#### Convolution/superposition algorithm

In commissioning photon beams computed via the convolution/superposition algorithm, as implemented on the Pinnacle^3^ treatment planning system, the calculated dose output (dose per monitor unit) is scaled to match measured dose output at a reference depth for a set of square field sizes. The vendor of the treatment‐planning system recommends a reference depth of 10 cm because at that depth, electron contamination is essentially absent, and the entire dose is due to photons. This is the same depth at which photon beam calibration measurements are taken in the TG51 protocol for clinical dosimetry.^6^ Output factors, relating the dose output for a set of square field sizes at the reference depth to the dose output for a reference field size at the same depth, are determined for calculated fields and compared with measured output factors. The reference field is conventionally taken to be a 10 cm×10 cm field. The ratio of the field‐size‐dependent measured output factor to the calculated output factor is used as a field‐size‐dependent correction factor that relates the calculated dose output to the measured dose output for the reference field size at the reference depth. Separation of the measured output factor into a calculated output factor and a field‐size dependent correction factor is analogous to the separation of the measured output factor into the Sp and Sc factors.^7^ When the dose for a beam of arbitrary field size and shape is computed, the measured collimator‐setting dependent output factor is multiplied by the ratio of the computed dose output for the beam to the computed dose output for the reference beam at the reference depth to obtain the true dose output for the desired beam. The number of monitor units is then determined by dividing the prescription dose by the dose output at the reference point.

### Monitor unit test set

In order to compare monitor units calculated via the radiation treatment‐planning system with monitor units obtained via point dose calculations, an extensive test set was developed. This test set was designed to sample a wide range of field sizes, shapes, and depths for isocentric treatments and is summarized in Table I.

**Table I:** Collimator settings, field sizes, and SSDs of fields comprising the monitor unit test set. Field widths greater than 15 cm were not available for 60° wedged field.

Open fields					
Collimator (cm×cm)	Field size (cm×cm)	SSD (cm)	Collimator (cm×cm)	Field size (cm×cm)	SSD (cm)
4×4	4×4	96	5×9	5×9	96
4×4	4×4	88	5×9	5×9	88
5×5	5×5	92	14×6	14×6	92
5×5	5×5	84	14×6	14×6	84
6×6	6×6	96	7×22	7×22	96
6×6	6×6	88	7×22	7×22	88
8×8	8×8	92	26×8	26×8	92
8×8	8×8	84	26×8	26×8	84
10×10	10×10	96	9×29	9×29	96
10×10	10×10	88	9×29	9×29	88
12×12	12×12	92	29×9	29×9	92
12×12	12×12	84	29×9	29×9	84
15×15	15×15	96	5×7	4×6	96
15×15	15×15	88	5×7	4×6	88
18×18	18×18	92	11×11	10×9	92
18×18	18×18	84	11×11	10×9	84
20×20	20×20	96	17×17	16×12	96
20×20	20×20	88	17×17	16×12	88
24×24	24×24	92	21×29	19×27	92
24×24	24×24	84	21×29	19×27	84
28×28	28×28	96	20×20	10×20	96
28×28	28×28	88	20×20	10×20	88
30×30	30×30	92	20×20	20×10	92
30×30	30×30	84	20×20	20×10	84
Wedged fields (15°, 30°, 45°, 60°)					
Collimator (cm×cm)	Field size (cm×cm)	SSD (cm)	Collimator (cm×cm)	Field size (cm×cm)	SSD (cm)
4×4	4×4	96	5×7	4×6	92
4×4	4×4	88	5×7	4×6	84
5×5	5×5	92	11×11	10×9	96
5×5	5×5	84	11×11	10×9	88
6×6	6×6	96	17×17	16×12	92
6×6	6×6	88	17×17	16×12	84
8×8	8×8	92			
8×8	8×8	84			
10×10	10×10	96			
10×10	10×10	88			
12×12	12×12	92			
12×12	12×12	84			
15×15	15×15	96			
15×15	15×15	88			
18×18	18×18	92			
18×18	18×18	84			
20×20	20×20	96			
20×20	20×20	88			

Treatment fields, as identified in Table I, were set up on a three‐dimensional computed tomography (CT) representation of a water phantom. Beams were computed on each of the two treatment‐planning systems, and monitor units were computed to deliver a specified dose to isocenter. Monitor units were compared with those computed using central‐axis depth dose data. These steps were repeated for each treatment machine for which beams were commissioned.

## RESULTS AND DISCUSSION

**Figure 1:**
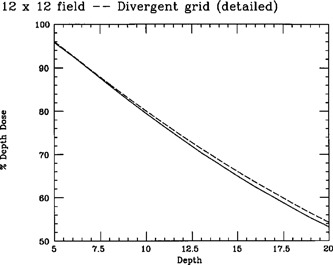
Comparison of measured (solid line) central‐axis depth dose for a 12 cm×12 cm field at 100‐cm SSD vs calculated (dashed line) central‐axis depth dose for a 12 cm×12 cm field at 80‐cm SSD and corrected to 100‐cm SSD using the Mayneord *F* factor.

In initial tests, monitor units computed using the FFT‐convolution algorithm differed from those computed using central‐axis depth dose data by as much as 2.5%. This difference was greater than our quality assurance acceptance criterion of 2%, and the discrepancy was systematic. This discrepancy was the result of the inability of our system to calculate the dose accurately for SSDs other than 100 cm. This discrepancy can be observed in Fig. 1. In this figure, the FFT convolution algorithm was used to calculate the central‐axis depth‐dose distribution for a 12 cm×12 cm field for an 18‐MV photon beam (Clinac 2100‐C, Varian Associates, Inc., Palo Alto, CA) at an SSD of 80 cm. These central‐axis depth doses were then converted to doses for an SSD of 100 cm using the Mayneord *F* factor, where they were compared with measured central‐axis depth doses for a 12 cm×12 cm field at an SSD of 100 cm. As can be seen in Fig. 1, calculation and measurements differed by up to 2%.

Examination of the implementation of the FFT convolution algorithm uncovered an error in the implementation. The error in calculation can be explained by noting that the primary photon fluence was calculated in a divergent fan‐line coordinate system. This coordinate system was selected because in a divergent fan‐line coordinate system, it is easy to calculate the primary fluence via ray tracing the primary fluence travels away from the radiation source in the direction of the fan lines. The dose‐spread kernels are also aligned along the fan lines, ensuring the correct spatial relationship between fluence and fan lines. However, in a divergent coordinate system, the calculation grid points are not equally spaced. Errors were caused in the convolution calculation because the FFT convolution algorithm requires equally spaced grid points.

One solution to this problem is to map the fan‐line coordinate system onto a Cartesian coordinate system. Calculation grid points then lie at uniform intervals, and one may then proceed with the FFT convolution. This is, in fact, what has been done to generate the dose distributions whose central‐axis depth doses are illustrated in Fig. 1. The problem with this approach, however, is that the dose‐spread kernels are not spatially invariant in the fan‐line coordinate system. Fan lines are placed more closely together when closer to the radiation source, thus dose‐spread kernels appear larger when closer to the radiation source. In fact, the central‐axis depth dose distribution for the 12 cm×12 cm field at 80‐cm SSD, calculated in this manner, and corrected to 100‐cm SSD, was identical to that of the measured dose distribution for a 15 cm×15 cm field. This is precisely the field size that would be seen by the rays that define the primary fluence if the rays were nondivergent.

In order to correct this problem, the software was modified to calculate the photon fluence in the fan‐line coordinate system but then transform the fluence into a Cartesian coordinate system before convolving it with the dose‐spread kernels. In this coordinate system, the kernels are spatially invariant, so the FFT convolution is accurate. A small error was introduced because the dose‐spread kernels were no longer aligned with the fluence, but this error was found to be negligible.

**Figure 2:**
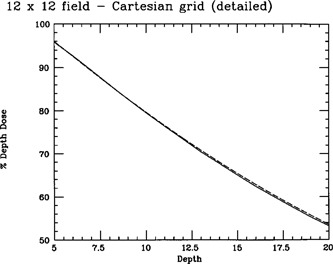
Comparison of measured (solid line) central‐axis depth dose for a 12 cm×12 cm field at 100‐cm SSD vs calculated (dashed line) central‐axis depth dose for a 12 cm×12 cm field at 80‐cm SSD and corrected to 100‐cm SSD using the Mayneord *F* factor. Primary fluence is computed on a divergent fan‐line grid and transformed to Cartesian coordinates prior to convolution.

The accuracy of the central‐axis depth‐dose calculation using the transformed fluence is illustrated in Fig. 2. As this figure illustrates, the change in coordinate system reduced the difference between calculation and measurement to less than 0.6%. Calculated monitor units matched monitor units obtained via central‐axis depth dose data to within our 2% acceptability criterion; moreover, the discrepancies were no longer systematic.

In the second example, we performed the beam calculations on the Pinnacle^3^ treatment‐planning system, setting up beams for the open fields and each wedge. Beams were queued, and the doses were computed. For most fields, treatment‐planning system calculations agreed with point dose calculations to within our quality assurance criterion of 2%, but discrepancies of up to 5% were observed for wedged fields, especially for 60° wedged fields. In the process of analyzing the cause for the discrepancies, we repeated the dose computation but reversed the order of the beams for calculation, i.e., we began computing with the 60° wedged fields and ended with the open fields. We were surprised to find different values for the monitor units. On this second pass, the monitor units for the 60° wedged fields agreed more closely with the point dose calculations, while monitor units for the open fields showed greater discrepancies. It appeared that the order in which calculations were performed affected the values of the monitor units calculated using the treatment‐planning system. This discrepancy was reported to the vendor of the treatment‐planning system.

Within a week, the vendor identified the cause of the problem. When applying the convolution model to a polyenergetic beam, one properly needs to integrate over the energy spectrum as well as over the three spatial dimensions. In the convolution/superposition algorithm, the energy integration is replaced by energy‐averaging the incident fluence as well as the dose‐spread array.^5^ The energy averaging of the dose‐spread array is a time‐consuming component of the dose computation, because it must be computed at every dose‐calculation point. In order to accelerate the calculation, the treatment‐planning system computes an energy average of the dose‐spread array for the first beam, and uses it for all beams of the same energy in the treatment plan. The energy‐averaged dose‐spread array is reasonably constant over a large range of field sizes, because the energy spectrum in the beam model is relatively independent of field size.^8^ Thus the approximation used by the treatment‐planning system vendor is reasonable under most clinically applicable circumstances. However, the differences in model spectra between an open field and a 60° wedged field are sufficient that to use the same dose‐spread array for both fields can cause an error of a few percent in the dose output.

After the vendor corrected this error in the implementation of the convolution/superposition algorithm, we resumed testing of the monitor unit calculations. With beam‐specific energy‐averaged dose‐spread arrays, monitor units were correctly calculated independent of the order in which the beams were computed. For square fields, monitor units calculated using the treatment‐planning system agreed with point dose monitor units to well within the 2% accuracy criterion. However, we still observed a systematic difference between the two sets of monitor unit calculations. In all cases, monitor units computed using the treatment‐planning system were greater than those computed using a point dose calculation, typically by 0.5% to 1.0%. Although the differences were within our accuracy criterion, it was still necessary to investigate a systematic discrepancy.

**Figure 3:**
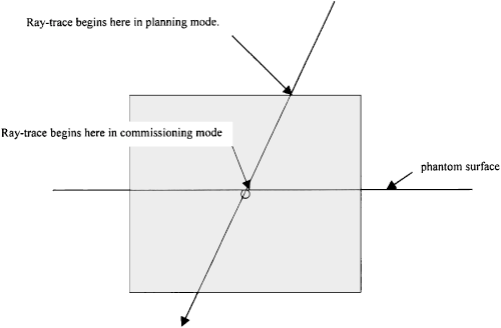
Illustration of ray tracing through CT voxel. In the beam‐commissioning mode ray‐tracing began at the true surface of the phantom while in the treatment planning mode, ray tracing began at the proximal surface of the CT voxel.

We believe the systematic discrepancy may be due to differences in the manner in which ray tracing is done in the beam‐commissioning mode from the way it is done when doses are computed in the treatment‐planning mode. In the beam‐commissioning mode, ray tracing is done from the true surface of a water phantom to dose calculation points. In the treatment‐planning mode, no phantom surface is explicitly defined; rather, ray tracing is begun at the proximal surface of the first voxel whose CT value corresponded to anything other than air. Because the true surface of the phantom is generally taken to be at the center of the voxel, the increased length of the radiological path caused by tracing from the proximal surface of a voxel led to a small increase in the depth of the dose calculation point and a corresponding increase in monitor units required to deliver the dose prescription. Figure 3 may help to clarify the differences in ray tracing. When dose calculations were performed in the treatment planning system using an option that ray‐traced from a hypothetical phantom surface defined to be at the central‐axis SSD, the systematic discrepancy was removed from the monitor unit calculations. The water phantom used in the monitor unit calculations tests has a voxel dimension of 2.5 mm. Thus the thickness of half a voxel is approximately 1.25 mm, which is likely to account for an error of approximately 0.5%.

In our clinical treatment planning, we elect to ray‐trace through the three‐dimensional CT array rather than ray‐trace from a presumed surface at central‐axis SSD. Consequently, we increased the value of the machine output (dose per monitor unit) entered into the treatment planning system by approximately 0.5% to remove the systematic discrepancy from the monitor unit calculations.

Monitor units calculated for elongated fields remained unacceptably greater (>2%) than those obtained from point dose calculations. Moreover, the calculated values depended on the orientation of the elongated field to a greater extent than was found by measurement. The calculated values also depended on the location of the collimators. When the collimators defining the narrow dimension of the field were closer to the phantom, the discrepancy between calculation and measurement was greater than when the collimators defining the wide dimension of the field were closer to the phantom. When we set both sets of collimators at the same distance from the source, the orientation dependence was removed. We therefore concluded that the cause of the discrepancy had to do with the modeling of the radiation coming from the collimators of the linear accelerator.

Within the beam model, the contribution to the dose most directly affected by the geometry of the collimators is the scatter from the flattening filter. In the treatment‐planning system, this scatter is modeled by adding to the in‐air photon fluence a contribution calculated by convolving a Gaussian distribution with a unit mask whose dimensions are identical to those of the collimator‐defined field.^9^ In the beam commissioning process, the height and width of the Gaussian are varied in order to fit the “tails” of the off‐axis profiles.^8^ By setting the Gaussian height to zero, the collimator‐dependent discrepancies in the monitor unit calculations could be significantly decreased. When this was done, however, the tails of the computed off‐axis profiles were unacceptably flatter than the tails of the measured profiles. A compromise was therefore necessary in which the height of the Gaussian was increased to a point where it reasonably modeled the tails of the off‐axis profiles yet gave acceptable monitor unit calculations for elongated fields while the width was kept at values previously determined by auto‐modeling.^8^

## CONCLUSIONS

In the several examples cited in this paper, we have demonstrated that testing the ability of the treatment‐planning system to calculate monitor units can help us identify subtle errors in the implementation of a dose‐calculation algorithm and in the data sets used in the dose model. In practical applications, the user of the treatment planning system cannot correct errors in the coding of the dose‐calculation algorithm as we were able to do with COPPERPlan. However, by performing an extensive series of tests with a commercial treatment planning system, we were able to identify errors in the system and provide the vendor with significant information to assist them in determining the cause of their coding errors. In reality, no code is completely error free,^10^ it is one of the responsibilities of the user to identify errors in a treatment‐planning system and report these errors to the vendor.^11^

Even if the implementation and coding of a dose‐calculation model is correct, it must be recognized that any model is an approximation to reality. Fitting model parameters for one aspect of the dose‐calculation model, such as off‐axis profiles, may compromise the accuracy of the dose calculation elsewhere, such as the calculation of monitor units. Furthermore, by understanding differences in the dose computation in various components of the treatment planning system, the user is able to make modifications in the beam data in order to generate a more accurate dose calculation. Commissioning a beam model represents a set of compromises, and all aspects of the beam computation must be analyzed to determine the extent of compromise necessary.

The tests that are described in this paper are relatively easy to set up and require approximately four to six hours to compute and analyze. Once the tests are set up, they may be stored and repeated with other beam models when additional beams are being commissioned. The time spent in performing these tests is small compared to the confidence in the dose calculation algorithm that performing these tests can instill in the user.
